# Medical students’ perceptions and confidence in their ability to apply nutrition principles in clinical practice

**DOI:** 10.15694/mep.2020.000211.2

**Published:** 2021-03-17

**Authors:** Kirsty Lennon, Fiona Muir

**Affiliations:** 1 University of Dundee

**Keywords:** nutrition, medical education, medicine, undergraduate

## Abstract

This article was migrated. The article was marked as recommended.

**Background:**Due to the rising rates of malnutrition, which can adversely affect health, doctors must be competent in addressing nutrition concerns in practice. This study explored medical students’ perceptions and confidence in applying nutrition principles in practice: nutrition assessment, patient counselling, and interventions.

**Methods:** A small scale exploratory case study was conducted using semi-structured interviews with eight undergraduate medical students. An inductive thematic analysis was carried out. Documentary analysis was completed using policy and Medical School curriculum documents to review nutrition-related text in terms of the learning outcomes for nutrition education.

**Results:** The findings highlight aspects which influenced students’ nutrition practice for patient assessment and intervention: students’ experience of nutrition both in education and practice, roles, importance of nutrition, concerns regarding application, barriers, and nutrition theme teaching. Documentary analysis results showed that the General Medical Council (GMC) and Dundee Medical School curriculum addressed higher level learning outcomes but students felt they were not achieving them.

**Conclusions:**This study identifies factors which contribute to students’ confidence in applying nutrition principles in practice with particular emphasis on nutrition curriculum and managing nutrition concerns in practice. It offers suggestion for curriculum review and development.

## Introduction

Malnutrition is defined as a serious condition that happens when a diet does not contain the right amounts of nutrients. It means ‘poor nutrition’ and can refer to: under-nutrition - not getting enough nutrients, or over-nutrition - getting more nutrients than needed ( NHS, 2020). It is an increasing problem in healthcare and can both cause illness, or be a consequence of illness.

Undernutrition can affect up to 3 million people in the United Kingdom at any one time, and can result in longer hospital stays and increased health and social care costs ( BAPEN, 2011; Baxter *et al.*, 2018). Acute illness can lead to malnutrition as it is a hypercatabolic state, where activation of acute inflammatory proteins, increased metabolic rate, and oxidation of fuel sources, can result in altered protein, carbohydrate, and fat metabolism ( Sharma, Mogensen and Robinson, 2019). One study demonstrated that the prevalence of malnutrition in intensive care units ranges from 38% to 78% ( Lew *et al.*, 2017). However, adequate nutrition support can improve response to illness and reduce nutrition-related complications ( Sharma, Mogensen and Robinson, 2019). Thus, timely assessment and intervention is important in preventing malnutrition in hospitals.

By contrast over-nutrition and obesity, defined as a body mass index (BMI) of more than 30kg/m ^2^, is a significant risk factor for the development of non-communicable diseases ( Purnell, 2000; Hill, Wyatt and Peters, 2012; Mozaffarian, Rosenberg and Uauy, 2018). Within Scotland alone 65% of adults aged 16+ are overweight, 29% are obese ( Scottish Government, 2017). Obesity contributes to four out of the five leading causes of death in Scotland (National Records of Scotland, 2017). However, appropriate nutritional strategies can be used to prevent, treat, or manage severity of symptoms in some diseases e.g. type two diabetes ( Hark, Deen and Morrison, 2014; Wass and Owen, 2014). Therefore, healthcare professionals should be able to identify and counsel patients where nutritional status may result in adverse health outcomes.

The importance of nutrition in healthcare is recognised in undergraduate medical training by the General Medical Council (GMC), who oversee medical practice and training in the UK, and the Academy of Medical Royal Colleges (AoMRC), who set standards for training across the 23 Royal Colleges and Faculties in the UK and Ireland. The GMC’s Outcomes for Graduates (2018) suggest that newly qualified doctors should be able to, assess and appropriately manage circumstances where poor nutrition contributes to illness; apply, in practice, scientific principles and knowledge of nutrition; discuss the role and impact of nutrition in healthcare ( General Medical Council, 2018b). All UK medical schools have until 2020 to implement the GMC ‘Outcomes for Graduates’ 2018 ( General Medical Council, 2018b). At the time of this study the GMC ‘Outcomes for Graduates’ 2018 was introduced into years one to three of the medical curriculum whereas the ‘Outcomes for Graduates’ 2015 remained in use for students in the later years to provide continuity in their education.

The AoMRC formed an Intercollegiate Group in Nutrition (ICGN) who developed core curriculum outcomes for undergraduate medical training, to aid medical schools in understanding the broader GMC outcomes ( House of Commons, 2011; ICGN, 2013b, 2013a). These nutrition-related outcomes, from two leading healthcare bodies, signify the importance of nutrition education in undergraduate medical training. Hence, medical schools should deliver nutrition education to achieve these recommendations.

Medical nutrition education has been scrutinised by media sources, who claim that medical students and healthcare professionals lack the skillset or confidence to apply the principles of nutrition in practice ( Womersley and Ripullone, 2017; Dillon, 2018; Rupy, 2018). Sunguya *et al.* (2013) found that doctors avoid nutrition counselling in practice because of inadequate nutrition knowledge. Similarly, Perlstein *et al.* (2016) found that less than 20% of senior medical students reported confidence in their knowledge of nutrition-related management guidelines for common chronic diseases, such as cardiovascular disease. Nutrition training interventions, for example, multi-disciplinary workshops and lectures delivered in primary care and the undergraduate medical curriculum, which aim to improve the communication around nutrition with patients, were found to increase participants’ nutrition knowledge, counselling skills and attitude towards the importance of nutrition in healthcare ( Schlair *et al.*, 2012; Ball *et al.*, 2014). Thus, current literature suggests that nutrition in medical education is inadequate and that more nutrition teaching could help resolve this concern.

This study aimed to establish fourth- and fifth-year medical students’ confidence in their nutrition knowledge and skills, and their perceptions of nutrition education, in light of the learning outcomes identified within the nutrition curriculum.

Objectives:

• To explore medical students’ confidence in their knowledge of nutrition principles and the skills required to apply them to clinical practice, and their perceptions of a doctor’s role in addressing nutrition concerns in clinical practice.
• To understand medical students’ experience of their nutrition education including their experiences of observing and practicing nutrition principles in clinical practice.
• To identify any perceived additional learning needs regarding nutrition in the undergraduate medical curriculum.

## Methods

An exploratory case study method ( Yin, 1993) was utilised within one Medical School in Scotland. A qualitative approach was chosen as it is more suitable for understanding a person’s experiences; this case aimed to understand students’ experience of the nutrition curriculum ( Saldaña, 2011). A case study was deemed the most suitable study design as it allows for studying a group of people where the phenomenon of interest exists ( Swanborn, 2010). This study explored the case of fourth- and fifth-year medical students registered on the MBChB programme at Dundee Medical School during the 2018/19 academic year.

### Study setting and participant sampling

Fourth- and fifth-year medical students at Dundee Medical School (n= 293) were invited to take part via email and written informed consent was gained prior to the interview. This cohort of students had completed, an estimated, 26 hours of nutrition teaching throughout years one to three of their five-year MBChB programme. They had started hospital and community clinical attachments with the opportunity to begin applying their knowledge and skills in practice. Participants were recruited using convenience sampling.

### Data collection

Data source triangulation was used in this study, by employing interviews and documentary analysis, allowing for different perspectives of the phenomenon to be explored and compared ( Barbour, 2008).

Semi-structured face to face individual interviews were conducted to explore students’ confidence in their nutrition knowledge and skills, and to understand their experiences of nutrition teaching in the classroom and clinical environment. The interviews were audio recorded and transcribed verbatim by the researcher. Identifiable information was removed from the interviews and each participant was given a code to ensure anonymity and confidentiality. Interviews were conducted with all the participants who volunteered. ‘Member checking’ was performed post interview by emailing the participants a copy of their interview transcript to ensure that it was an accurate representation ( Birt *et al.*, 2016).

Documentary analysis was carried out to review nutrition-related text within policy and Dundee Medical School curriculum documents to identify the outcomes which students are expected to achieve in nutrition education. This included: General Medical Council (GMC) ‘Outcomes for Graduates (Tomorrow’s Doctors)’ (2015) and ‘Outcomes for Graduates’ (2018); ICGN ‘Undergraduate Medical Curriculum on Nutrition’ ( ICGN, 2013b) and ‘Accompanying Notes’ ( ICGN, 2013a); Dundee Medical School’s nutrition curriculum including the learning and teaching materials consisting of workbooks and lecture slides. GMC Outcomes 2015 and 2018 were included as both were embedded in the medical curriculum at the time of this study.

### Data analysis

The method as ascribed by Braun and Clark (2006) was used for data analysis ( Braun and Clarke, 2006). Each interview was examined and coded independently, and the number and types of codes were derived from the data ( Spencer, Ritchie and O’Connor, 2003).

**Figure 1.**
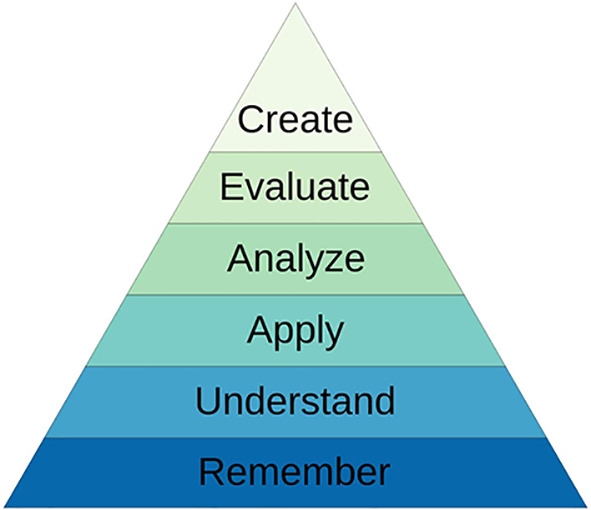
Revised Bloom’s Taxonomy

The documents were analysed for nutrition-related text. The medical school teaching materials were viewed and the learning outcomes from lectures and workshops were extracted and analysed to identify the level of Bloom’s taxonomy ( Figure 1) they addressed i.e. knowledge, understanding, application and so on ( Krathwohl, 2002). The same method was used to analyse the GMC and ICGN outcomes. The purpose of analysing these documents was to identify what nutrition teaching should occur following GMC and ICGN guidance and what nutrition teaching actually occurs at Dundee. This information was compared with the transcribed interview data, to gauge if students had received these learning opportunities and whether they had achieved the nutrition competencies of the GMC ( Table 1).

**Table 1.** Example, using nutritional assessment, of how document analysis and interview findings were compared

GMC Outcomes for Graduates 2015	GMC Outcomes for Graduates 2018	ICGN UK Undergraduate Curriculum in Nutrition	IGCN Medical Undergraduate Curriculum in Nutrition - Accompanying Notes	Dundee Nutrition Theme Curriculum Learning Outcomes	Interview quotes
“NutritionalAssessment: Making an assessment of the patient’s state of nutrition. This includes an evaluation of their diet; their general physical condition; and measurement of height, weight, and body mass index.” [p.12].	Do not explicitly provide outcomes relating to nutritional assessment or BMI.	“Assess nutritional state and risk for malnutrition, using physical examination, body mass index (BMI) and waist circumference; perform valid measurements of height, weight and waist circumference, calculate BMI and interpret the results” [p.8]	“It is important for all students to acquire the practical skills relating to the assessment of nutritionalstate either in clinical skills training or at the bedside, integrated with history taking and physical examination.”[p.2]	Year 1 - Nutritional Screening workshop: Demonstrate valid measurement of height and weight. Demonstrate valid use of surrogate measures of height and weight.	*“I’ve observed people using MUST scores, to just kind of assess the persons’ nutritional status and see what needs to be done from that.”* P4Y4 *“I’ve been asked to do MUST scores... and BMIs“* P6Y4

### Ethics

This study was approved by the School of Medicine Research Ethics Committee prior to data collection in 2019 (application number: 128/18). Ethical considerations were applied throughout the research ( British Educational Research Association, 2018). Participants were given codes P1 to 8 alongside a descriptor of which year group they belonged to (Y4 and Y5), to provide anonymity and protect confidentiality.

## Results

### Interview Findings

#### Participant characteristics

Eight interviews were conducted with fourth year (n=five) and fifth-year medical students (n=three). The participants were undergraduate students, three males and five females between the ages of 21-23 years. Thematic analysis produced six main themes which are evidenced by participants’ quotes.

#### Theme 1. Participants’ experience of nutrition education

Participants’ educational background and experience of nutrition was explored to understand what nutrition education they had received within the medical curriculum and out with. Two participants had completed a Student Selected Component, an optional module to extend their experience and interests beyond the core curriculum, in breast and infant feeding. This involved revision of concepts from the medical curriculum and some new knowledge.

My final SSC that I did most recently was breast and infant feeding... it was mostly revision...- P7Y4

Students’ had varying experience of observing and performing nutrition-related skills in the clinical environment, including using their communication skills to discuss nutrition concerns with patients. The majority of participants had observed nutrition practice (i.e. nutrition assessment, counselling, and intervention), although questioned its contribution to their knowledge and understanding of nutrition. Half of the participants had performed nutrition-related tasks including insertion of a nasogastric tube in the healthcare environment. However, there was a lack of understanding if a Malnutrition Universal Screening Tool (MUST) score ( BAPEN, 2011) or Body Mass Index calculation was deemed as performing a skill.

I’ve been asked to do MUST scores a few times and BMIs if that counts - P5Y4

All participants believed they had the relevant communication skills to address nutrition concerns in practice although only half felt confident in doing this. Participants said their confidence depended on the patients’ needs and expectations.

My confidence will come with time, when I’m more confident in my skills I’ll feel more confident talking to somebody about their weight. - P3Y5

#### Theme 2. Perceptions on the role of the doctor and the dietician

Participants were unsure how much nutrition knowledge and expertise they were expected to have as doctors. They were unsure of their role as future doctors in providing nutritional care when compared to a dietician’s role, but believed it is a doctor’s duty to address nutrition concerns in practice. Participants said they realised in the latter phase of the curriculum that it was a doctor’s duty to address nutrition concerns in practice; as they began to appreciate the importance of dietary issues in healthcare.

In 1 ^st^/2 ^nd^/3 ^rd^ year there was a perception that this is something that doesn’t really apply to us... dieticians do most of it. - P1Y5

We need to know how we can provide the dietician care without being a dietician. - P8Y4

I think it is our duty to start talking more about the prevention side of medicine and public health aspects of it. - P7Y4

Participants thought they lacked nutrition knowledge and experience in the ‘specialist’ areas of nutrition, e.g., selecting and calculating rates of feed. Although they had grasped general nutrition concepts, they suggested it wasa surface understanding where knowledge was more basic.

A potential barrier could just be lack of knowledge about it, so maybe you’re able to say to the person about their weight... but then you’re not really able to take it any further... you don’t know as much about it. - P4Y4

It’s [nutrition] very much a specialist subject and I find it quite difficult to follow. -P1Y5

Lack of exposure, especially with regards to NG tube feeding...we don’t really have anything to do with that, so I wouldn’t know much about it. - P5Y4

#### Theme 3. Perceptions of the importance of nutrition

Participants identified the importance of nutrition in healthcare although this realisation came later in the curriculum from gaining exposure to nutrition concerns in the clinical environment. They indicated, that previously, they did not appreciate the worth of nutrition education (in years one to three) and, due to their focus on the end of year exams, they viewed nutrition as less important.

If you’ve got time commitment to revising all the stuff we’ve done in 4 years it’s not top of the list. - P6Y4

There was that perception previously that it wasn’t as important as some of the other stuff we were being taught. - P2Y5

#### Theme 4. Concerns about application

All participants had a desire to understand how their early years teaching lends itself to clinical work in later years; to apply the knowledge and skills from their learning, for example, in real clinical scenarios. Participants had some understanding of nutrition but did not know how they would form an appropriate management plan for a patient.

I just feel that it’s a very challenging subject and without the clinical relevance it’s a bit harder to grasp. - P1Y5

There’s maybe slight gaps with how to apply it practically ... [for example] a ward scenario or a patient-case. - P2Y5

#### Theme 5. Perceived barriers

When asked if there were barriers to addressing nutrition concerns in practice, participants reported nutrition as a sensitive topic. Participants acknowledged that diet and weight are sensitive subjects which discouraged them from raising the issue. As medical students they held a view that they did not have time to build rapport with patients, lacked authority as a medical student, and inferred that it would be inappropriate for them to raise the issue.

It is a sensitive issue, you don’t want to offend people... that would probably be the only thing that would stop me bringing it up. - P3Y5

When we’re [medical students] on the ward, the patients are doing more benefit to us than we are to them, so we don’t really want to offend them in any way which quite often bringing up their weight does. - P6Y4

#### Theme 6. Views on the nutrition curriculum

There were differing opinions about the nutrition teaching in years 1-3 of the programme; half of the participants thought the teaching equipped them to deal with nutrition concerns in practice. This included communication skills, raising awareness of the importance of nutrition, basic nutrition principles, and weight management for overweight or obese individuals. Less developed concepts included the clinical application of nutrition, their role in managing nutrition as future doctors, and undernutrition including different forms of feeding and nutritional supplements.

It’s [teaching] made me more conscious of nutrition as a concept and that it is important for doctors and nurses to broach the subject with patients. - P4Y4

I think maybe it would be good to have... in fourth-year a ...session on nutrition again... and relate it more practically. - P8Y4

### Documentary analysis findings

#### Policy and guidance documents

From analysing the documents, differences were found between the GMC expected outcomes and the ICGN competencies. These included outcomes relating to ‘knowledge’ and ‘skills’. The ICGN curriculum is not compulsory for medical schools to follow. Thus, students within UK medical schools which have not implemented the ICGN guidance may not achieve the relevant level of nutrition knowledge or skill.

Image: Nicoguaro CC/BY

Utilising Bloom’s taxonomy ( Figure 1) to review the outcomes relating to knowledge, the GMC require students to achieve higher levels of learning namely application. The GMC refer to ‘apply’ nutrition knowledge in practice ( General Medical Council, 2015, p. 2), whereas, the ICGN only address the remembering and understanding levels of Bloom’s taxonomy.

Prior to graduation students are required to be competent in nutrition skills, including, nutritional assessment as noted in Appendix 1: ‘Practical Procedures for Graduates’, in ‘Outcomes for Graduates’ 2015 ( General Medical Council, 2015). Notably, this requirement was removed from ‘Outcomes for Graduates’ 2018. By contrast, the ICGN list of nutrition-related skills, is more comprehensive than the GMC. However, unlike the GMC outcomes, the ICGN is not compulsory, so it is less likely that all medical schools will have implemented these outcomes ( ICGN, 2013b). A potential gap would therefore exist in students’ learning of nutrition-related skills at schools where they have not implemented the ICGN curriculum; skills no longer required by the GMC.

Unlike the limited nutrition outcomes set by the GMC ‘Outcomes for Graduates’, the ICGN curriculum, which guides medical schools to implement an appropriate nutrition curriculum *,* provides direction on the implementation of nutrition teaching within the teaching environment ( ICGN, 2013a). Hence, it is a useful document for medical schools to follow when designing and implementing a nutrition blueprint.

Comparing GMC’s ‘Outcomes for Graduates’ 2015 and 2018 there is now more emphasis on promoting wellbeing and patient self-care by improving diet. However, the new practical skills list does not include nutritional assessment ( General Medical Council, 2019). ‘Outcomes for Graduates’ 2015 referenced the ICGN in an appendix titled ‘related documents’, but now state in a supplementary document linked to ‘Outcomes for Graduates’ 2018, that they do not formally endorse them ( General Medical Council, 2018a). Whilst there may be more outcomes relating to nutrition knowledge for patients, in the form of self-care and lifestyle advice, the requirement for medical students to have nutrition assessment skills including evaluating diet and body mass index has been removed.

### Dundee Nutrition Curriculum

When reviewing the medical school nutrition curriculum, teaching occurred across the first three years of the five-year medical curriculum and the majority of teaching was delivered by lecture or small group workshops. Most learning outcomes were at the remembering and understanding level of Bloom’s taxonomy ( Figure 1). However, almost all workshops had at least one outcome that was of a higher level, such as application, to integrate some higher order learning into the nutrition teaching. Furthermore, a workbook which lists 112 competencies that fourth- and fifth-year students should achieve by the end of their clinical rotations was also reviewed. Three areas were identified relating to nutrition: nutrition, nutritional assessment, and nasogastric tube insertion ( University of Dundee, 2018). No additional guidance is provided for the tasks nutritional assessment and nutrition. Therefore completion of these tasks rely on the student or supervisor’s understanding of the term nutrition. Thus, the medical school nutrition teaching outcomes appear to seek to deliver higher level nutrition teaching in years one to three and outlines the competencies students are expected to learn and achieve, whereas the competencies in subsequent years appears to be less defined.

## Discussion

In concordance with other studies, participants in this study appreciated the need for nutrition education but were less confident in their nutrition knowledge and clinical skills ( Vetter *et al.*, 2008; Sunguya *et al.*, 2013; Ball *et al.*, 2016; Perlstein *et al.*, 2016). Students had the communication tools necessary to discuss nutrition with patients, as stated by P8Y4: “confident enough to raise the weight issue and we’ve been taught how to communicate it sensitively“. However, medical student status, lack of knowledge and experience, and the sensitivity of the subject were contributing factors towards why students were less confident in addressing nutrition in practice. Furthermore, students wanted to understand how their early years teaching lends itself to clinical work in later years. This is similar to the findings of Hardman *et al.* (2015) and Mogre *et al.* (2018).

At the time of this study, the GMC expected students to apply scientific principles of nutrition in practice, discuss the impact of nutrition on health, and carry out nutrition-related skills by the time they graduate ( General Medical Council, 2015). To complement this, the learning outcomes of the nutrition teaching materials at Dundee Medical School sought to address higher levels of learning, including application. However, students did not feel they had achieved these outcomes. Reasons for this included their perceived lack of knowledge, clinically related teaching opportunities, and not having a clear understanding of their future role in managing nutrition concerns.

Participants felt that they developed a better appreciation of the doctor’s role in nutrition practice in the later years of study from observing nutrition assessment and management in the clinical environment. The early curriculum teaching, delivered by dieticians, may account for why students were less able to appreciate the clinical relevance of the nutrition teaching in years one to three or the doctor’s role in nutrition practice. Hence, they did not make the connection between the teaching content and its application to clinical practice. As adult learners, they are required to be internally motivated and need to understand why they need to know something ( Kauffman and Mann, 2014).

This presents the medical school with an opportunity to review curriculum alignment, where the learning outcomes match the lesson content and affiliate with forms of assessment to encourage deep, as opposed to surface learning ( Walsh, 2007; Ali, 2018). Ali (2018) suggests that, ‘alignment is about student’s realization to take full participation in the responsibility of their own learning’ (p.73). Hence medical students should better understand the relevance and value of the nutrition teaching in years one to three. There could be a benefit in integrating more nutrition content within the end of year exams, as participants noted there was a need. Assessment is known to drive learning, thus, when there is limited nutrition material in an exam, students focus on the subjects that feature more prominently, to achieve the highest grade, which is similar to the opinions of the participants in this study ( Friedman *et al.*, 2010; Harrison and Wass, 2016; Pugh and Regehr, 2016). This could enhance the students’ motivation to learn about nutrition and retention of knowledge of the nutrition teaching.

Most participants acknowledged the doctor’s role in addressing nutrition concerns in practice but were uncertain how much nutrition expertise they required, and their role in managing patient concerns. Currently, the nutrition teaching is predominantly delivered by dieticians. This may explain the limited student understanding about their role as future doctors. Promotion by medical clinicians complementary to the dietician’s role could be useful to highlight the doctors’ role and subsequent engagement from the dietician and other services, similar to clinical practice ( Kushner *et al.*, 2014). Teaching from other healthcare professionals could reinforce the clinical relevance of nutrition teaching.

### Limitations

The participants of this small scale study were not easily accessible due to the busy academic calendar of the selected student cohort, thus convenience sampling was used for recruitment ( Etikan, 2016). While there was incomplete data saturation due to the relatively small number of participants who agreed to participate in the study, this was the number available via the recruitment. Although data saturation was incomplete, common patterns emerged from the interview data.

This case study was conducted in a single medical school. It is not intended that the findings represent the general opinion of all medical students. However, this study illustrates the student experience of nutrition education, which is similar to those from other institutions in Ghana, Australia, and the USA ( Hardman, Miller and Shah, 2015; Perlstein *et al.*, 2016; Mogre *et al.*, 2018). These findings add to the current body of literature on medical nutrition education from a Scottish perspective. This could guide effective nutrition teaching in other medical schools.

## Conclusion

The results of this study are relevant to modern clinical practice owing to the rising rates of nutrition-related disease. Modern healthcare has a focus on prevention rather than treating the consequences of poor nutrition, as recognised by the GMC and the Academy of Medical Royal Colleges ( ICGN, 2013b; General Medical Council, 2018b). It is important that students can assess, counsel, and intervene to improve patients’ nutrition and avoid the potential adverse outcomes. The findings from this study could support the medical school in reviewing the alignment of the nutrition learning outcomes. Greater clinical application could enable students achieve the learning outcomes and develop a range of skills and experiences in preparation for their future clinical responsibilities.

## Take Home Messages

• Students’ attitudes towards nutrition have a significant impact on their learning. Clinically relevant teaching and emphasising the importance of nutrition early in the medical curriculum have been suggested by students as methods of changing students’ attitudes.
• Nutrition teaching should be delivered by an inter-professional team to ensure medical students are clear about their role now and in the future, the role of the dietician and others.
• The medical school may wish to review the alignment of the learning outcomes and its clinical application to enable students to achieve higher levels of Bloom’s learning outcomes regarding nutrition.

## Notes On Contributors

Kirsty Anne Lennon is a Final Year Medical Student at School of Medicine, University of Dundee. In 2019, she completed an intercalated Bachelor of Medical Sciences (BMSc) degree in Medical Education at the University of Dundee. ORCID ID: https://orcid.org/0000-0001-6903-2965

Dr Fiona Muir EdD, MEd, SFHEA, BA, RSCN, RGN Senior Lecturer, Centre for Medical Education, School of Medicine, Dundee within undergraduate and postgraduate education. A highly experienced dual-professional: academic and nurse registrant with extensive experience. Research interests: reflective practice, inter-professional & interdisciplinary education, professionalism, doctor as teacher, multi-cultural education. ORCID ID: https://orcid.org/0000-0002-8636-7094
