# Supplementary material for: Medical students’ perceptions and confidence in their ability to apply nutrition principles in clinical practice
Source: MedEdPublish (2016). 2021 Mar 17;9:211. Originally published 2020 Sep 29. [Version 2] doi: 10.15694/mep.2020.000211.2 (PMC10699404; doi:10.15694/mep.2020.000211.2)
Supplement: Peer review history

# Reviewer report: Reviewer response for version 2

**DOI:** 10.21956/mep.20282.r31490

**Published:** 2021-06-12

**Authors:** Ken Masters

**Affiliations:** 1 Sultan Qaboos University

This review has been migrated. The reviewer awarded 4 stars out of 5 The paper is Version 2 of a small qualitative study examining medical students’ perceptions and confidence in their ability to apply nutrition principles in clinical practice.The authors have appropriately addressed the reviewers’ major concerns of Version 1, and have produced a final version of the paper that is a useful contribution to the field. This is in spite of the fact (as acknowledged by the authors) that data saturation was not reached. A good and useful read, and a study that many at other medical schools should consider performing.

Reviewer Expertise:

NA

No decision status is available

# Reviewer report: Reviewer response for version 2

**DOI:** 10.21956/mep.20282.r31489

**Published:** 2021-03-23

**Authors:** Megan Anakin

**Affiliations:** 1 University of Otago

This review has been migrated. The reviewer awarded 4 stars out of 5 Thank you for publishing this revised version of your article. I appreciate the time you spent addressing the comments and suggestions from reviewers. This article has now been strengthened and presents the data you collected and analysed in way that provides insights into the perceptions and confidence in the ability of medical students to apply nutrition principles in clinical practice. The revisions allow an international reader to appreciate the work you have done in the UK context to address this important area of the medical curriculum and health of the population. The conclusions you draw from your study are now presented effectively. This article will be very helpful to simulate conversations with colleagues about the paucity of learning outcomes and opportunities related to nutrition in the medical curriculum at my institution. I look forward to reading about the next steps of your research in this area.

Reviewer Expertise:

NA

No decision status is available

# Reviewer report: Reviewer response for version 1

**DOI:** 10.21956/mep.19142.r27672

**Published:** 2020-12-12

**Authors:** Megan Anakin

**Affiliations:** 1 University of Otago

This review has been migrated. The reviewer awarded 3 stars out of 5 Like the two previous reviewers, I appreciated this article about medical students’ perceptions and confidence about their ability to apply nutrition principles in clinical practice. The warrant for the study is well-established in the introduction. The aim of the study is clearly stated and is shown to be related to three course objectives.To further support the choice of using an exploratory case study approach, please consider expanding the single sentence about the study design to convince that sampling a small number from population of 293 year 4 and 5 medical students will best address the study aim. There is insufficient explanation given in the methods to convince the reader that the 8 students sampled (with only 3 students from year5) are appropriate to address the research question. To strengthen the results, please also consider reporting the findings of the document analysis so the reader can see how the interview data were compared. In the results section, please consider revising how themes are presented so that the unit of analysis is clear to the reader. Please clarify that the themes represent the perceptions and confidence of the group of 8 participants rather than individuals within the group. Please consider omitting the words “Some participants said…” and “Several realised…”. Please either give the participants pseudonyms or random alphanumerical identity codes (such as P1Y4, P2Y4, …) so the reader can see that the quotations do not all come from one Y4 and one Y5 student. Right now, there is no way to see if the voices of all participants are presented in the results or not. Please consider renaming the first theme since it is the same as the second objective and does not help the reader appreciate the students’ perception and confidence about that curriculum objective. Likewise, please reconsider the names of the other themes so they more accurately cue the reader to how the study participants expressed their perception and confidence about ability to apply nutrition principles in clinical practice. To support the suggestion about presenting the findings of the documentary analysis, please consider replacing the current Figure 1 with a Table that compares the GMC outcomes and the ICGN competencies with the findings of the interview analysis. In the second sentence of the discussion, please support the claim with an example from the results. In the limitations section, the statement about incomplete data saturation contradicts the statement made in the methods section that “data saturation occurred”. To better express and explain this idea, please consider reading: Jacqueline Low (2019) A Pragmatic Definition of the Concept of Theoretical Saturation, Sociological Focus, 52:2, 131-139, DOI: 10.1080/00380237.2018.1544514In the take home messages section, please consider revising the points so they more accurately reflect the study’s main findings. In the first point, please consider avoiding the use of the word significant because it is usually associated with statistical results.I look forward to reading a revised version of this article.

Reviewer Expertise:

NA

No decision status is available

# Reviewer report: Reviewer response for version 1

**DOI:** 10.21956/mep.19142.r27671

**Published:** 2020-10-03

**Authors:** Shaoting Feng

**Affiliations:** 1 The First Affiliated Hospital of Sun Yat-sen University

This review has been migrated. The reviewer awarded 4 stars out of 5 It is an interesting and important study on nutrition education for undergraduate medical students in UK. I think this study is also inspiring for the nutrition education for undergraduate medical curriculum of in China, as the rates of nutrition-related disease have been continually rising these days. It reminds us that we should emphasize the importance of nutrition education for medical students. Maybe further study is needed with a larger number of participants coming from more medical schools, if we want to know the general opinion of more medical students, which is quite important but really difficult.

Reviewer Expertise:

NA

No decision status is available

# Reviewer report: Reviewer response for version 1

**DOI:** 10.21956/mep.19142.r27673

**Published:** 2020-10-02

**Authors:** P Ravi Shankar

**Affiliations:** 1 American International Medical University

This review has been migrated. The reviewer awarded 4 stars out of 5 This is an interesting manuscript about nutrition education in a medical school in the United Kingdom. Nutrition is an area which does not get ‘’much’’ attention in the medical school curriculum. The authors describe teaching-learning in nutrition all through the undergraduate course which is encouraging. One of the challenges as mentioned by the authors is the dominant role of dieticians in teaching nutrition during the early years so students are not clear about the exact role of doctors regarding nutrition. I am happy to note that one of the authors is a medical student. Some of the terms are UK-centric and may need explanation for international readers. I was not aware about the Academy of Medical Royal Colleges. In the Caribbean I found the online nutrition course being offered by the University of North Carolina to be a useful supplement to student learning. Fundamental nutrition and advise on food and how to eat well did not receive the emphasis they require in medical school. The methodology is well described and is comprehensive. I enjoyed reading this well-written and presented manuscript.

Reviewer Expertise:

NA

No decision status is available
